# From Controllers to Multimodal Input: A Chronological Review of XR Interaction Across Device Generations

**DOI:** 10.3390/s26010196

**Published:** 2025-12-27

**Authors:** Hyejin Kim, Sukwon Lee, Changgu Kang

**Affiliations:** 1 AI & Big Data Research Division, The Seoul Institute, 89-10 Seomun-ro, Jung-gu, Seoul 04516, Republic of Korea; hyejin.kim@si.re.kr; 2 Multimedia IT Engineering, Gangneung-Wonju National University, Wonju-si 26403, Republic of Korea; sukwonlee@gwnu.ac.kr; 3 Department of Computer Science and Engineering, Gyeongsang National University, Jinju-si 52828, Republic of Korea

**Keywords:** XR interaction techniques, user study analysis, chronological survey

## Abstract

This study provides a chronological analysis of how Extended Reality (XR) interaction techniques have evolved from early controller-centered interfaces to natural hand- and gaze-based input and, more recently, to multimodal input, with a particular focus on the role of XR devices. We collected 46 user study–based XR interaction papers published between 2016 and 2024, including only studies that explicitly defined their interaction techniques and reported quantitative and/or qualitative evaluation results. For each study, we documented the XR hardware and software development kits (SDKs) used as well as the input modalities applied (e.g., controller, hand tracking, eye tracking, wrist rotation, multimodal input). These data were analyzed in relation to a device and SDK timeline spanning major platforms from the HTC Vive and Oculus Rift to the Meta Quest Pro and Apple Vision Pro. Using frequency summaries, heatmaps, correspondence analysis, and chi-square tests, we quantitatively compared input modality distributions across device generations. The results reveal three distinct stages of XR interaction development: (1) an early controller-dominant phase centered on the Vive/Rift (2016–2018), (2) a transitional phase marked by the widespread introduction of hand- and gaze-based input through the Oculus Quest, HoloLens 2, and the Hand Tracking SDK (2019–2021), and (3) an expansion phase in which multisensor and multimodal input became central, driven by MR-capable devices such as the Meta Quest Pro (2022–2024). These findings demonstrate that the choice of input modalities in XR research has been structurally shaped not only by researcher preference or task design but also by the sensing configurations, tracking performance, and SDK support provided by devices available at each point in time. By reframing XR interaction research within the technological context of device and SDK generations—rather than purely functional taxonomies—this study offers a structured analytical framework for informing future multimodal and context-adaptive XR interface design and guiding user studies involving next-generation XR devices.

## 1. Introduction

Over the past several years, extended reality (XR) technologies have rapidly transitioned from prototype-oriented systems to available consumer platforms. This technological shift has fundamentally reshaped interaction methods in XR environments, enabling a broad spectrum of input modalities ranging from traditional controller-based interactions to hand gestures, eye tracking, body movements, and multimodal combinations of these inputs [1,2,3,4]. Advancements in XR devices and software development kits (SDKs) have further facilitated the development of natural, intuitive, and context-adaptive interaction techniques, driving continuous expansion and diversification in XR interaction research.

Early XR interaction research was predominantly shaped by the 6-degree-of-freedom (6-DoF) controllers introduced with first-generation head-mounted displays (HMDs) such as the HTC Vive and Oculus Rift. Studies during this period mainly focused on performance-oriented evaluations—including accuracy, selection performance, and manipulation efficiency—and examined controller-based techniques such as raycasting and direct manipulation as primary research targets [5,6,7,8,9,10,11,12]. A major shift occurred with the release of the Oculus Quest and HoloLens 2 in 2019 and the public launch of the Hand Tracking SDK in 2020, which collectively accelerated the widespread adoption of hand-tracking and gaze-based inputs [13,14,15,16,17,18,19,20,21,22]. More recently, the introduction of devices equipped with high-precision sensors and mixed reality capabilities—such as the Meta Quest Pro (2022) and Apple Vision Pro (2024)—has established multimodal interaction as a central research trend, integrating inputs from the hands, gaze, wrist, voice, and other sensing channels [3,23,24,25,26,27].

Along with these technological shifts, a growing number of survey studies have examined XR interaction techniques. Chen et al. (2024) [28] provided an overview of XR research with a focus on VR interface design principles and interaction categories, while Zhang et al. (2016) [29] conducted an early comprehensive review of HCI techniques in VR environments. In addition, several partial surveys have focused on specific modalities such as hand tracking, gaze tracking, and gesture-based input, and recent quantitative comparisons have examined differences between controller- and hand-tracking–based techniques or between direct and indirect manipulation methods [30].

However, most existing surveys center on functional classifications or technology-specific categorizations, and they provide limited explanations of how the evolution of XR hardware has shaped broader temporal trends and structurally influenced changes in input techniques. In particular, few studies have examined how the emergence of major devices and SDKs—such as the HTC Vive and Oculus Rift (2016) [31,32], Oculus Quest and HoloLens 2 (2019) [33,34], the Hand Tracking SDK (2020) [35], and the Apple Vision Pro (2024) [36]—has influenced the trajectory of XR interaction research. Specifically, it remains unclear how the introduction of these technologies triggered shifts in input modalities and at which points certain techniques began to gain widespread adoption. While existing surveys provide valuable snapshots of interaction techniques at specific moments in time, they offer limited chronological analysis of how technological advancements themselves have driven paradigm shifts in XR interaction research.

This study aims to address this gap by providing a chronological analysis of how XR interaction research has evolved over the past nine years, with a particular focus on the technological advancements of XR devices and SDKs. To this end, we collected 46 user study–based XR interaction papers published between 2016 and 2024 and systematically examined changes in interaction modalities and research trends in relation to the technological environment at the time of each publication.

The main contributions of this study are as follows: (1) We present a chronological analysis of XR interaction techniques from 2016 to 2024, illustrating a paradigm shift from controller-based interactions to natural hand- and gaze-based inputs and, more recently, to multimodal interaction; (2) We construct a timeline of major XR device and SDK developments and analyze how key technological milestones have influenced the direction of interaction technique research; (3) Based on 46 XR interaction studies, we summarize the evolution of input modalities and derive correlations between technological advancements and shifts in research focus; (4) We discuss future directions for XR interaction research and propose research challenges necessary for advancing multimodal and context-adaptive interaction design.

By offering an integrated technological and chronological perspective on the past nine years of XR interaction development, this study provides an essential foundation for understanding future research directions and the broader implications of XR technology evolution.

## 2. Methodology

This study investigates how advancements in XR devices and software development kits (SDKs) have shaped the evolution of XR interaction research from 2016 to 2024. To this end, we collected and analyzed XR interaction studies published during this period and examined the resulting trends from a chronological and technology-centered perspective. The literature search was conducted in the first half of 2025. Although the manuscript was prepared and submitted later in the year, the review intentionally focuses on studies published between 2016 and 2024 to maintain a consistent chronological scope aligned with major XR device and SDK generations.

This systematic review was guided by the PRISMA guidelines to structure the literature search, screening, eligibility assessment, and study selection process. As this study is a systematic review of prior literature, the analysis is guided by research questions rather than formal hypotheses.

To systematically examine the evolution of XR interaction research in relation to technological advancements, this review was guided by the following research questions:

- **RQ1.** How have XR devices and SDKs evolved from 2016 to 2024, and how can this evolution be characterized into distinct technological stages?
- **RQ2.** How have interaction input modalities and techniques in XR research changed across these stages during the period from 2016 to 2024?
- **RQ3.** What technological constraints and affordances introduced by XR devices and SDKs appear to influence the adoption of interaction techniques, and what implications do these trends suggest for future multimodal XR interaction design?

Because XR interaction research is distributed across multiple academic domains, we performed literature searches using four major scholarly databases: IEEE Xplore, ACM Digital Library, SpringerLink, and Google Scholar. Search keywords included a broad range of terms associated with XR interaction techniques and input modalities, such as “VR interaction,” “XR interaction,” “hand tracking,” “controller interaction,” “gesture input,” “raycasting,” “gaze interaction,” “wrist rotation,” and “text entry in VR.” The search period was set to 2016–2024, aligning with the commercialization of the HTC Vive and Oculus Rift, which marked the beginning of modern 6DoF-based VR interaction research [5,37].

Studies were included if they satisfied the following inclusion criteria:

- **IC1.** The study conducted actual user experiments in XR (VR/AR/MR) environments.
- **IC2.** The interaction input modality or technique was clearly described.
- **IC3.** Quantitative and/or qualitative results evaluating interaction performance or user experience were reported.
- **IC4.** The study was published in a major international conference or journal in the XR or HCI domain.

Studies were excluded if they met any of the following exclusion criteria:

- **EC1.** The study was not focused on XR interaction (e.g., purely technical or non-interactive XR systems).
- **EC2.** The study was published outside the target period (2016–2024).
- **EC3.** The study was a duplicate record across multiple databases.
- **EC4.** The full text was unavailable or did not provide sufficient methodological details for analysis.

This review does not involve the recruitment of new human participants. Instead, the unit of analysis is previously published XR interaction studies that themselves report user experiments. Participant-related characteristics are therefore considered only insofar as they contextualize the findings reported in individual studies, rather than being analyzed as primary variables in this review.

A total of 46 studies were ultimately selected. For each study, we extracted the publication year, XR devices used (e.g., HMDs, controllers, hand-tracking modules), the SDK employed, the applied input modalities (e.g., controller, hand tracking, gaze, wrist rotation), characteristics of the proposed interaction technique, and the reported experimental results.

As this study is a systematic review, no new experiments were conducted. Instead, the analysis focused on aggregating and examining interaction input data reported in prior XR studies using quantitative methods, including frequency analysis, correspondence analysis, and chi-square tests, to identify relationships between XR devices and interaction modalities.

To capture the technological evolution of XR devices and SDKs, we constructed a chronological timeline centered on major technological milestones in the XR ecosystem, including the HTC Vive (2016) [31], Oculus Rift (2016) [32], Windows Mixed Reality (2017) [38], Oculus Quest (2019) [33], HoloLens 2 (2019) [34], the Hand Tracking SDK (2020) [35], Meta Quest Pro (2022) [39], and the Apple Vision Pro (2024) [36]. After arranging the 46 selected studies by publication year, we identified the prominent input modalities and interaction techniques characteristic of each period and analyzed the relationships between technological advancements and emerging research trends.

Based on the analysis, we further structured the evolution of XR interaction research into three stages: (1) the early stage (2016–2018), during which controller-based interactions dominated; (2) the transitional stage (2019–2021), marked by the widespread adoption of hand-tracking and gaze-based input technologies; (3) the expansion stage (2022–2024), characterized by the rise of multimodal interactions integrating hand, gaze, wrist, voice, and other sensor-based inputs [23,40,41]. This staged analysis provides an integrated interpretation of how advancements in XR devices and SDKs have driven paradigm shifts in XR interaction research.

## 3. Evolution of XR Devices and SDKs

Shifts in XR interaction research have been influenced less by individual research interests or task design preferences and more directly by the technological constraints and capabilities afforded by XR hardware and software development kits (SDKs) at each point in time. In this context, advancements in XR devices and SDKs have served as both the origin of new input modalities and key turning points that expanded interaction technique research.

Table 1 summarizes the chronological evolution of major XR devices and SDKs from 2016 to 2024, highlighting key interaction-related capabilities introduced at each stage.

This section summarizes major technological developments in XR devices and SDKs from 2016 to 2024 and examines their chronological implications for XR interaction research. Based on this timeline, XR interaction research exhibits three major developmental stages. The following section provides a detailed examination of the characteristic research directions that emerged during each period.

### 3.1. Emergence of Commercial 6DoF HMDs and the Establishment of Controller-Based Interaction Research (2016–2018)

The commercial release of the HTC Vive and Oculus Rift CV1 in 2016 marked the beginning of modern XR interaction research. These devices provided 6DoF spatial tracking and dedicated motion controllers, establishing a standardized environment for studying core 3D interaction tasks such as spatial manipulation, pointing, and selection [5,37,42]. The Lighthouse-based external tracking system of the Vive offered high positional precision, enabling studies focused on accuracy-driven tasks, including Fitts’ law–based selection experiments, analyses of 3D approach behavior, and bimanual manipulation [6].

During this period, available SDKs were primarily designed around controller input events, leading researchers to develop techniques leveraging trigger pressure, button events, and controller pose information. Consequently, XR interaction studies between 2016 and 2018 largely centered on evaluating the performance and efficiency of traditional 3D UI techniques such as raycasting, direct manipulation, and menu navigation [7]. These studies later served as a baseline for comparisons with hand-tracking–based input, providing quantitative foundations for analyzing performance differences across interaction techniques [43].

### 3.2. Commercialization of Hand and Eye Tracking and the Expansion of Natural Input Research (2019–2021)

The introduction of the Oculus Quest and HoloLens 2 in 2019 marked a significant technological and conceptual shift in XR interaction research. The Quest, as a fully standalone HMD, substantially reduced the cost and complexity of setting up experimental environments, enabling studies to be conducted across a wider range of user contexts. In contrast, the HoloLens 2 offered precise joint-level hand tracking, which facilitated research on near-field manipulation and natural gesture-based input [15,16,18,19,44,45].

The release of the Oculus Hand Tracking SDK in 2020 further accelerated this shift by enabling interaction entirely without controllers, giving rise to new interaction metaphors such as pinch-based selection, mid-air button activation, and finger-based UI elements [46,47]. As devices equipped with eye tracking became more widely available, research on gaze-assisted pointing and combined gaze–hand input also grew rapidly [1,13,14,20].

Ultimately, the expansions introduced by SDKs during this period were not merely incremental feature additions but shifts that reshaped task design paradigms in interaction experiments. These advancements enabled research to move beyond purely mechanical task execution toward studies incorporating user behaviors (e.g., gestures, gaze) and cognitive processes such as attention [17,48,49].

### 3.3. Integrated Multimodal Sensing and the Expansion of MR-Based Interaction (2022–2024)

In 2022, the Meta Quest Pro became the first consumer XR device to integrate hand tracking, eye tracking, and facial expression tracking within a single system, enabling controlled experiments on multimodal input. In addition, its high-resolution passthrough and scene-understanding capabilities facilitated research that blended VR and AR paradigms, accelerating the development of mixed reality (MR) interaction techniques [3,50,51,52]. Within MR environments, new research topics emerged, including spatial user interfaces, environment-aware interfaces, and interactions grounded in real-world objects. Studies investigating pseudo-haptics for weight perception also appeared during this period [53,54,55].

The growing adoption of OpenXR further standardized device APIs, enabling more reproducible user studies and facilitating cross-device comparison experiments. Consequently, XR interaction research from 2022 to 2024 expanded toward multimodal input techniques that combine hands, gaze, voice, wrist rotation, and other sensing channels. Higher-level interaction concepts such as context-adaptive input, multimodal fusion interfaces, and environment-aware selection emerged as central research themes [4,23,24,27,40,56]. The release of the Apple Vision Pro in 2024 further solidified this shift by offering OS-level integration of hand, eye, and voice input, establishing a new standard for multimodal XR interaction.

### 3.4. Summary

Over the past nine years, advancements in XR devices and SDKs have significantly reshaped the trajectory of interaction technique research. From 2016 to 2018, 6DoF controller-based input—centered on the Vive and Rift—dominated quantitative performance evaluations. Between 2019 and 2021, natural input techniques based on hand and gaze emerged and expanded with the introduction of the Oculus Quest, HoloLens 2, and the Hand Tracking SDK. From 2022 to 2024, multimodal sensing–based interaction—integrating hand, gaze, voice, and facial tracking—along with MR-focused research became the primary direction, driven by devices such as the Quest Pro and Vision Pro. These shifts represent not merely improvements in hardware capabilities but technological turning points that have redefined problem formulations, input modeling perspectives, and the experimental design paradigms of XR interaction research.

## 4. Device-Centric Analysis of XR Interaction Inputs

XR interaction techniques have continuously evolved alongside advancements in XR hardware. In particular, changes in input modalities have had a direct influence on user experience in HMD–based XR systems. This section analyzes the evolution of interaction input methods with a focus on major XR devices and examines how these developments are reflected quantitatively in published research.

**Figure 1:**
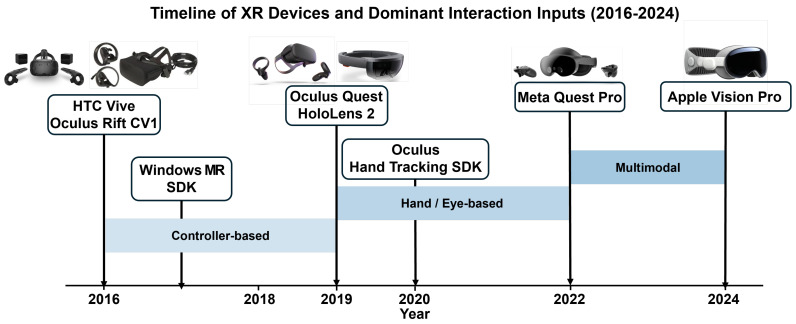
Evolution of major XR interaction input modalities across device generations (2016–2024).

Figure 1 illustrates how device-specific transitions in XR systems are reflected in the distribution of interaction input modalities across the analyzed studies, consistent with trends reported in the literature.

As shown in Table 2, the period from 2016 to 2018 was dominated by controller-centric HMDs such as the HTC Vive and Oculus Rift, with most studies employing raycasting or direct-controller–based input techniques. After 2019, hand- and eye-based interaction methods expanded rapidly as devices such as the Oculus Quest, Leap Motion, and Tobii eye trackers became more widely adopted in research. Notably, since 2022, multimodal interaction techniques integrating two or more input channels have shown a clear increase compared with the 2016–2018 period, driven by the growing use of multisensor devices such as the Meta Quest Pro and HoloLens 2.

These shifts demonstrate the evolution of XR interfaces away from single-mode input toward more natural and intuitive user experiences. Furthermore, the dependence of input modality choice on device-specific capabilities and sensor configurations highlights the need for interaction strategies that account for device characteristics in the design of future XR systems.

**Figure 2:**
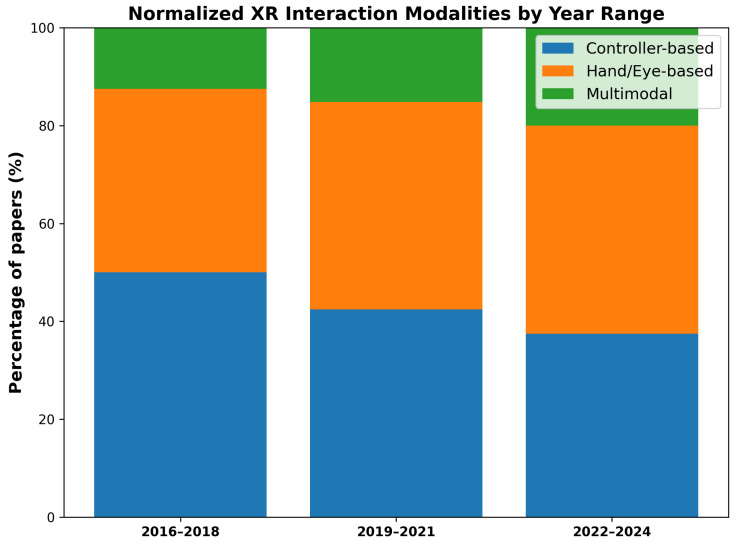
Normalized distribution of XR interaction input modalities across three time periods (2016–2018, 2019–2021, 2022–2024).

Figure 2 presents the same dataset normalized as relative proportions (%) across the three time periods. This visualization enables direct comparison of the relative prevalence of controller-based, hand/eye-based, and multimodal interaction techniques across periods with different numbers of studies, thereby highlighting structural shifts in XR input technologies rather than absolute publication counts. The results clearly illustrate a gradual transition in XR interaction—from controller-dominant methods in 2016–2018, to hand- and eye-centered techniques in 2019–2021, and further toward multimodal expansion in 2022–2024.

**Figure 3:**
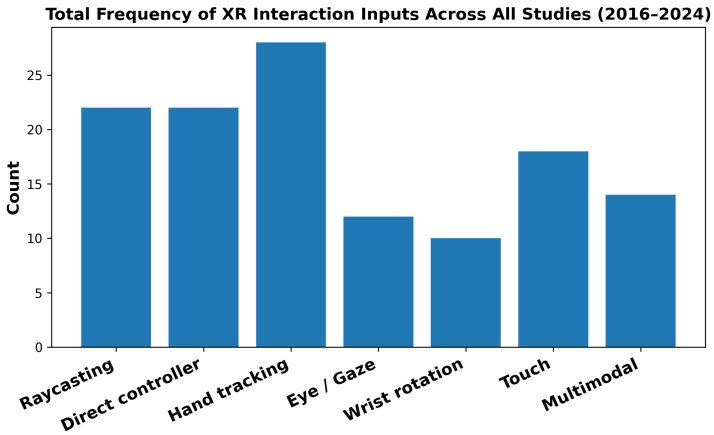
Total frequency of XR interaction input modalities across all reviewed studies (2016–2024).

Figure 3 summarizes how frequently each interaction input modality appeared across all 46 papers analyzed in this study, regardless of device type or time period. Hand-tracking was the most frequently used modality, while raycasting and direct-controller techniques were also employed at comparable rates. Touch-based interaction appeared with moderate frequency, and eye/gaze-based input and multimodal input, although less common, still accounted for a notable portion of the studies. Wrist rotation exhibited the lowest overall frequency. Overall, this distribution indicates that XR interaction research remains strongly oriented toward hand-based and pointing-centric interaction paradigms, even as newer modalities such as gaze-based and multimodal input continue to emerge.

While the temporal analysis presented earlier illustrates how XR technologies have evolved over time, it is equally important to understand which input modalities are predominantly associated with specific device generations. To this end, we analyzed the 46 XR studies published between 2016 and 2024 and categorized the distribution of input techniques according to the device generation used in each study.

**Figure 4:**
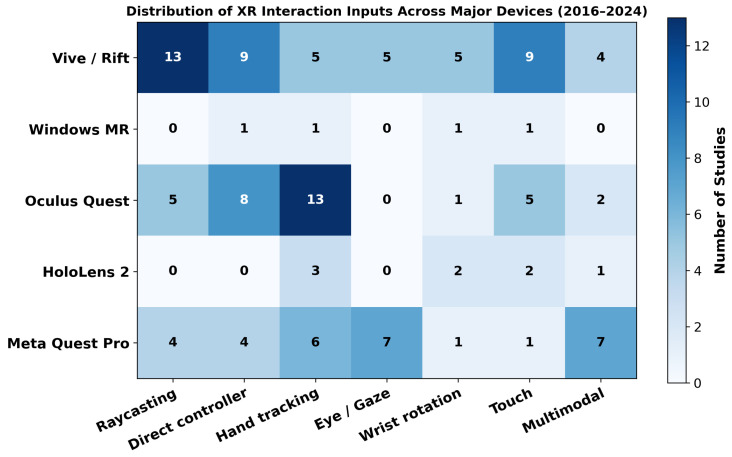
Distribution of XR interaction input modalities across major XR devices (2016–2024).

Figure 4 visualizes how frequently each interaction input modality—raycasting, direct controller input, hand tracking, eye/gaze input, wrist rotation, touch, and multimodal input—was utilized across studies involving major XR devices, including the Vive/Rift, Windows MR, Oculus Quest, HoloLens 2, and Meta Quest Pro. Although the Apple Vision Pro is a recent XR headset released in late 2024, its release timing resulted in no user study–based research employing the device within the 2016–2024 publication range.

In the early generation dominated by the Vive and Rift, raycasting, direct controller input, and touch-based interaction accounted for the majority of techniques used, indicating a strong reliance on controller-centered interaction paradigms. Research based on the Oculus Quest showed a substantial rise in hand-tracking techniques, driven by the device’s built-in support for controller-free hand input. Studies using the HoloLens 2 exhibited distinctive use of spatial gesture input, such as touch-like interactions in mid-air and wrist rotation, and showed minimal reliance on controller-based techniques typically found in VR systems.

Research leveraging the Meta Quest Pro demonstrated relatively high frequencies of eye/gaze input, hand tracking, and multimodal interaction, highlighting that modern XR devices support and encourage interaction designs integrating gaze, hand, and multiple sensing channels. Notably, multimodal input was most prominent in Quest Pro studies but also appeared to some extent in research using the Quest and Vive series, suggesting a gradual and broadening adoption of composite interaction techniques across the XR research landscape.

These findings confirm that device-specific sensor configurations, tracking capabilities, and SDK support directly shape the selection of input modalities and the overall direction of interaction research. Accordingly, future XR interface design should consider not only the performance of individual input techniques but also the technological characteristics and sensing infrastructure provided by each device when determining appropriate interaction strategies.

**Figure 5:**
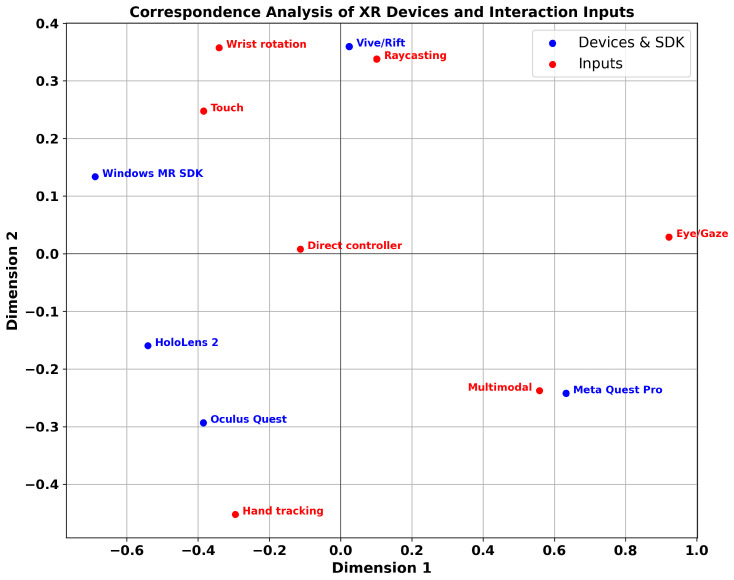
Correspondence analysis (CA) plot showing associations between XR devices and interaction input modalities across reviewed studies.

Figure 5 presents the results of a correspondence analysis (CA) visualizing the relationships between XR devices and interaction input modalities. In the 2D CA space, devices (blue points) and input modalities (red points) are plotted such that shorter distances indicate combinations that appeared more frequently together in the analyzed studies.

Overall, the Vive/Rift cluster near raycasting, direct controller input, and touch, reflecting that early XR research primarily relied on traditional controller-centric interaction techniques. In contrast, the Meta Quest Pro appears closest to eye/gaze input, hand tracking, and multimodal interaction, suggesting that modern XR devices actively support interaction paradigms grounded in gaze, hands, and combined sensing. This is consistent with the high proportion of multimodal and eye/gaze-based techniques observed in studies using the Quest Pro. Notably, multimodal input also appears to some degree in research involving the Vive/Rift and Oculus Quest, and accordingly, its CA position lies along the trajectory connecting these devices with the Quest Pro.

The Oculus Quest is positioned near hand tracking and direct controller input, illustrating the device’s role in popularizing both controller-free hand input and direct manipulation via controllers. HoloLens 2 appears relatively close to wrist rotation and touch, which aligns with its characteristic interaction style in spatial AR environments that emphasize gesture-based manipulation and wrist-centered input.

These CA visualization results quantitatively demonstrate that technological factors—such as sensor configurations, tracking capabilities, and SDK support—directly influence the selection of interaction input modalities in XR studies. Therefore, future XR interface design should adopt a strategic approach that considers not only the performance of individual input techniques but also the interaction affordances and constraints provided by each device.

To test whether input modality choices differ significantly across device generations, we conducted a chi-square test of independence using the device–input contingency table. The analysis yielded a chi-square statistic of 38.73 with 24 degrees of freedom and a *p*-value of 0.0291. Because this value is below the conventional significance threshold of 0.05, the result indicates a statistically significant association between XR devices and the input modalities adopted in the literature.

In other words, the choice of input modality varies systematically depending on the generation or type of XR device, and this pattern cannot be attributed to random variation. Instead, it reflects structural differences arising from device-specific technological characteristics and sensor configurations. These findings are consistent with the correspondence analysis results shown in Figure 5, reinforcing the notion that device-centered interaction design plays a critical role in shaping XR research practices.

## 5. Discussion

This study analyzed 46 user study–based XR interaction papers published between 2016 and 2024 to examine how advancements in XR devices and SDKs have structurally influenced the evolution of interaction input modalities and research trajectories. Recent XR interaction studies have increasingly explored novel, sensor-driven input techniques enabled by advances in XR hardware, such as eye tracking and hands-free interaction, reflecting a growing interest in multimodal and context-aware interfaces [57,58]. While many recent works focus on proposing and evaluating specific interaction techniques or systems, the present study situates these efforts within a broader, device-centered and longitudinal perspective.

The findings indicate that XR interaction research can be categorized into three stages: (1) An early stage (2016–2018) dominated by controller-based input centered on the Vive and Rift; (2) A transitional stage (2019–2021) during which natural hand- and gaze-based input gained widespread adoption with the introduction of the Oculus Quest, HoloLens 2, and the Hand Tracking SDK; (3) An expansion stage (2022–2024) characterized by multimodal input driven by the Meta Quest Pro and the broader emergence of MR-capable devices.

The correspondence analysis further revealed clear associations between XR device generations and input modalities. The Vive/Rift were closely linked to raycasting and direct controller input, while the Oculus Quest showed strong associations with hand-tracking–based techniques. The HoloLens 2 aligned with wrist-rotation and spatial touch-like gestures, and the Meta Quest Pro clustered near eye/gaze input, hand tracking, and multimodal interaction. These patterns suggest that the choice of input modality in XR research is not merely a function of researcher preference or task characteristics, but is structurally shaped by the sensing capabilities and SDK features provided by the devices available at the time.

The proposed device-generation-based framework offers practical implications for both XR interface design and experimental research. By accounting for the sensor configurations and SDK capabilities characteristic of each device generation, researchers and designers can more rationally select input modalities that are appropriate for a given XR platform. In addition, the framework provides a technical rationale for the inclusion or exclusion of specific interaction techniques in user studies, thereby improving the transparency and reproducibility of experimental design. Furthermore, in next-generation XR environments that natively support multimodal input, the framework highlights a shift beyond isolated performance comparisons toward interaction designs that adaptively combine or switch input channels according to task demands and contextual conditions.

However, this study has inherent limitations related to its temporal scope. Because the analysis covers literature published only up to 2024, experimental research using the Apple Vision Pro—released in late 2024—is not included. The Vision Pro represents the first consumer XR platform to offer OS-level integration of hand, eye, and voice input, enabling a richer form of multimodal interaction than previous HMDs. As Vision Pro–based user studies are expected to accumulate from 2025 onward, future findings may refine or extend the device generation–based interaction trajectory proposed in this study.

Another limitation is that the analysis focused primarily on input modalities. Advances in output and feedback modalities—such as haptic feedback, spatial audio, optical rendering techniques, and predictive model–based interaction—were not extensively considered. Similarly, device-level comparisons centered on input mechanisms, SDKs, and sensor configurations, without fully integrating multidimensional factors such as task variations, user characteristics, or cognitive load. Addressing these aspects will be essential for improving the generalizability of future XR interaction frameworks.

Despite these limitations, this study provides a structured analytical perspective by reframing XR interaction research within the technological context of device generations. This framework clarifies when and why major shifts in interaction techniques occurred and offers a foundation for understanding the mechanisms driving the evolution of XR interaction research.

**Figure 6:**
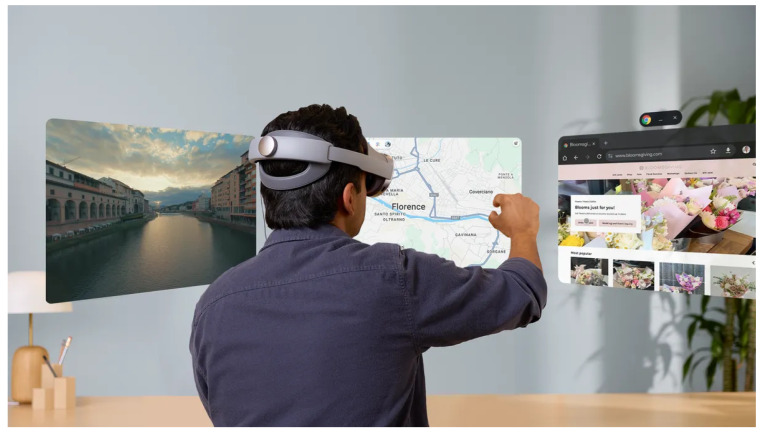
Samsung Galaxy XR headset showcasing integrated spatial interfaces. The device supports eye tracking, hand tracking, voice input, and MR passthrough, exemplifying the industry’s transition toward multimodal XR interaction. Image source: Google Blog (https://blog.google/intl/ko-kr/products/android-play-hardware/androidxr_galaxyxr/, accessed on 25 December 2025).

More recently, next-generation mixed reality devices such as Samsung’s Galaxy XR headset (Figure 6) have emerged, integrating eye tracking, hand tracking, spatial passthrough, and voice input within a single platform. The fact that these commercial XR devices now provide multisensor input as a default capability suggests that the trend toward multimodal interaction—identified in this study—is likely to accelerate even further. This indicates that XR interaction research will continue to evolve beyond performance comparisons of individual input techniques toward integrated multimodal interfaces that incorporate combinations of diverse input channels and support context-aware interaction.

## 6. Conclusions

This study examined 46 user study–based XR interaction papers published between 2016 and 2024 to investigate how technological advancements in XR devices and SDKs have shaped the evolution of interaction input modalities from a chronological perspective. The analysis revealed a clear progression in XR interaction techniques: from an early stage dominated by 6DoF controller-based input, through a transitional phase characterized by the widespread adoption of hand- and gaze-based input, to a recent expansion phase centered on multisensor, multimodal interaction. These shifts demonstrate that the evolution of XR interaction cannot be explained solely by comparing the performance of individual input techniques; rather, the sensing configurations, tracking capabilities, and SDK functionalities available at each point in time have structurally influenced research directions and interaction design paradigms.

The main contributions of this study are threefold. First, we provide a chronological synthesis of XR interaction research grounded in the evolution of commercial XR devices, offering a device-centered perspective on how technological milestones influence interaction design. Second, we quantitatively analyze device–input relationships using frequency analysis, correspondence analysis, and statistical testing, demonstrating systematic associations between XR devices and interaction modalities. Third, we propose a structured analytical framework that situates XR interaction techniques within device and SDK generations, enabling a more integrative understanding of past and emerging interaction trends.

Because this study considered literature published only up to 2024, user studies employing newer XR platforms such as the Apple Vision Pro were not included. As research on next-generation, multisensor XR devices is expected to grow rapidly, future work should extend this analysis to post-2024 literature to validate and refine the device generation–based interaction trajectory identified in this study. In addition, future research may expand the analytical scope beyond input modalities to incorporate technique-level categorizations, multimodal fusion strategies, and task-dependent input selection models. Addressing factors such as context-adaptive interaction, long-term usability, accessibility, and cognitive load will also be essential for developing comprehensive XR interaction frameworks.

Overall, this study provides a structured foundation for understanding the evolution of XR interaction technologies by situating them within the technological context of device and SDK generations rather than purely functional taxonomies. This perspective can inform future XR system design, device selection strategies, user study planning, and the development of robust multimodal interaction models.

## Figures and Tables

**Table 1:** Chronological evolution of major XR head-mounted devices and SDKs (2016–2024) and their key interaction-related features.

Device (Year)	Key Interaction-Related Features
HTC Vive (2016) [31]	A 6DoF VR system using Lighthouse-based external tracking; provided high-precision motion controllers.
Oculus Rift CV1 (2016) [32]	Equipped with Touch controllers supporting bimanual input such as direct manipulation and raycasting.
Windows MR SDK (2017) [38]	Introduced camera-based inside-out 6DoF tracking on HMDs, eliminating the need for external sensors.
Oculus Quest (2019) [33]	A fully standalone 6DoF HMD with complete inside-out tracking.
HoloLens 2 (2019) [34]	Enabled precise joint-level hand tracking and spatially grounded MR interactions.
Oculus Hand Tracking SDK (2020) [35]	Provided controller-free hand gesture, pinch, and finger-based input capabilities.
Meta Quest Pro [39]	Integrated eye tracking, face tracking, and enhanced hand tracking.
Apple Vision Pro (2024) [36]	Offered OS-level integration of hand, eye, and voice input with high-resolution passthrough for MR interaction.

**Table 2:** Number of XR papers by interaction modality and year range.

Year Range	Controller-Based	Hand/Eye-Based	Multimodal
2016–2018	4	3	1
2019–2021	14	14	5
2022–2024	15	17	8

## Data Availability

The original contributions presented in this study are included in the article. Further inquiries can be directed to the corresponding author.
